# Pro-domain processing of fungal effector proteins from plant pathogens

**DOI:** 10.1371/journal.ppat.1010000

**Published:** 2021-10-20

**Authors:** Megan A. Outram, Peter S. Solomon, Simon J. Williams

**Affiliations:** Research School of Biology, The Australian National University, Canberra, Australia; University of Maryland, Baltimore, UNITED STATES

## Introduction

Some secreted proteins and peptides are translated as precursors (pro-proteins/peptides) that require post-translational site-specific proteolysis to generate the mature, active molecule. Pro-protein convertases, a family of serine proteases found in the secretory pathway of fungi (and other eukaryotes), are typically responsible for these cleavage events and target secreted proteins with a wide range of physiologically important functions. We now know that some effector proteins from plant fungal pathogens are processed by pro-protein convertases during secretion and that this is important for effector virulence functions. Recent work suggests that this process is important for the maturation of an expanded set of effector proteins from a broad range of fungal plant pathogens.

## How are fungal effectors processed by proteases?

**Fig 1:**
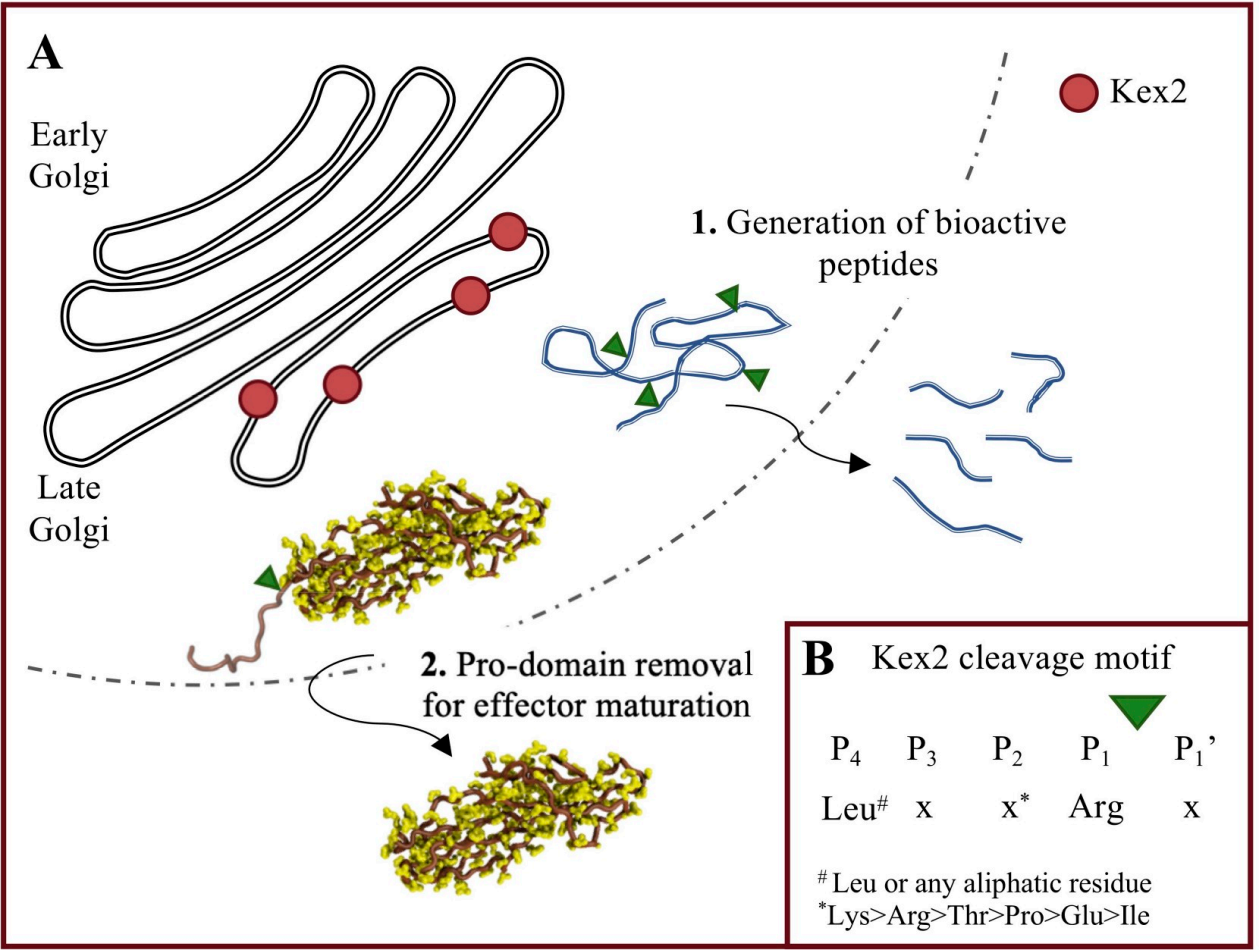
Fungal effector proteins and peptides are processed by a Kex2-like homologue prior to secretion. **(A)** Site-specific proteolytic processing of fungal effectors can be broadly classified in 2 classes. (1) Class 1: Precursor proteins cleaved by a Kex2-like protease releasing bioactive peptides (including repeat-containing effectors and RiPPs). (2) Class 2: K2PP effectors are produced with an N-terminal pro-domain, which is cleaved prior to secretion of the effector to produce the mature protein. SnTox3 from *Parastagonospora nodorum* (PDB: 6WES) is shown in cartoon form illustrating this process. **(B)** The cleavage motif of Kex2 protease, following typical protease nomenclature. P1 is almost exclusively an Arg, and cleavage occurs at the carboxyl end of P1. P2 preference is Lys, but there is capacity for significant variation (denoted as an x). The alternative top 5 ranked P2 amino acids (Arg, Thr, Pro, Glu, and Ile), as determined by Bevan and colleagues [21], are shown. P3 does not participate in direct interactions with Kex2 and is variable as denoted by x. P4 preference is a Leu but can also be any other aliphatic residue, including Ile, Val, Ala, or Pro. Ala, alanine; Arg, arginine; Glu, glutamic acid; Ile, isoleucine; K2PP, Kex2-processed pro-domain; Leu, leucine; Lys, lysine; Pro, proline; RiPP, ribosomally synthesised and post-translationally modified peptide; Thr, threonine; Val, valine.

Fungal effector proteins that are processed by proteases can be broadly separated into 2 classes (Fig 1A). The first class are effectors that function as bioactive peptides. Repeat-containing effectors have been identified in several fungal plant pathogens, the most well characterised are those originating from the maize pathogenic fungus *Ustilago maydis*. In *U*. *maydis*, 15 repeat-containing proteins have been identified. Of these, 8 appear to be processed into multiple short peptides by the calcium-dependent subtilisin-like serine protease Kex2 (kexin; E.C.3.4.21.61) [1]. One example, Rep1, is processed into multiple short peptides by Kex2 during secretion from the fungus. The generated peptides are biologically active and form tightly bound amyloid-like fibrils on the hyphal surface that repel water and facilitate the proper formation of aerial hyphae and hyphal attachment [2,3]. Additionally, some classes of ribosomally synthesised and post-translationally modified peptides (RiPPs) are known to be processed by a Kex2-like protease (recently reviewed by [4]). Examples include Victorin, a host-selective toxin produced by the oat pathogen *Cochliobolus victoriae* [5], and epichloëcyclins from *Epichloë festucae*, though their biological function remains unknown [6].

The second class of cleaved fungal effectors are processed via the removal of a pro-domain from the N-terminus of the effector [7] (Fig 1A). Effectors in this class were originally identified based on the presence of a canonical Kex2 protease cleavage motif and the absence of the proceeding N-terminal region of the protein (putative pro-domain) when isolated from source [7–12]. We recently confirmed that the effector SnTox3 from the wheat pathogen *Parastagonospora nodorum* is a pro-domain–containing effector. The secreted form of SnTox3 has the pro-domain removed, and the processed SnTox3 protein is more potent in activating Snn3-dependent cell death [7]. We showed that the specific removal of the pro-domain from SnTox3 could be achieved *in vitro* using recombinant Kex2 protein. We subsequently demonstrated other effectors (including ToxA from *P*. *nodorum* and secreted in xylem (SIX) effectors, SIX1, 4, and 6, from *Fusarium oxysporum* f. sp. *lycopersici*) possessed pro-domains that could also be removed by Kex2 *in vitro*, despite some of them possessing non-canonical Kex2 cleavage motifs. We defined these as Kex2 pro-domain–processed (K2PP) effectors and suggest that this class of effectors is more prevalent in fungal pathogens than previously thought.

## Is Kex2 responsible for effector protein pro-domain processing in fungi?

Kex2 protease homologues are likely responsible for fungal effector protein pro-domain processing during secretion. Kex2 was first identified from *Saccharomyces cerevisiae* to be essential for the maturation of the killer toxin precursors and alpha-factor mating pheromone precursors [13]. In yeast, Kex2 predominantly localises to the late *trans*-Golgi network [14], and an endocytic, prevacuolar compartment [15] to co-locate with proteins that are secreted via canonical secretion pathways.

A single copy of a Kex2 homologue has been identified in numerous fungal species, including pathogens of humans and plants [14,15]. We sought to directly link SnTox3 pro-domain processing with Kex2 by generating a Kex2 knock-out in *P*. *nodorum* but were unable to obtain transformants suggesting its requirement for viability in this fungus [7]. To date, a handful of successful Kex2 deletion mutants in fungi have been reported, including *S*. *cerevisiae*, and the human pathogenic fungi *Candida albicans* and *Candida glabrata* [16–19]. In fungal plant pathogens, successful silencing of Kex2 has only been reported in *Cryphonectria parasitica*, the causal agent of chestnut blight [20]. Collectively, these mutants suggest a role of Kex2 in fungal virulence. For example, reverse genetics approaches in *C*. *albicans* have demonstrated that Kex2 is required for two well-characterised virulence-related processes, hyphal formation and proteinase secretion [18,19]. In *C*. *glabrata*, reduced virulence was also observed in an *in vitro* epithelial model [17]. Despite this, in all instances, the Kex2 mutants displayed significant morphological phenotypes that affected growth and general viability, making it difficult to directly link Kex2 with fungal virulence and by extension effector processing.

The best evidence to implicate Kex2 in effector pro-domain processing is the presence of a cleavage motif. The Kex2 prototypical cleavage motif was originally described as a dibasic motif with a preference for lysine (Lys) or arginine (Arg) at P2 (following typical nomenclature for proteases; Fig 1B) and an invariant Arg at P1. Cleavage occurs at the carboxyl side of the P1 Arg [17,21,22]. While most of the selectivity towards the substrate arises through interactions at P2 and P1, recent studies suggest an expansion of this motif. There is a preference for leucine (Leu) or other aliphatic residues at position P4 [23,24]. No direct interaction between the residue at P3 and Kex2 occurs, but kinetic analysis suggests that an aspartate (Asp) at this position is unfavourable [22]. Additionally, and most relevant to K2PP effector classification, is the capacity for variation at P2. Using an *in vivo* assay to assess mating competence in yeast, Bevan and colleagues demonstrated that residues other than Arg and Lys can be accommodated at the P2 position [21] (Fig 1B).

Data supporting an extended Kex2 motif and a greater tolerance of variable residues at the P2 position have ultimately altered the search parameters used to identify putative Kex2-targeted proteins. This work has resulted in the identification of an expanded number of Kex2 processed secreted proteins [25,26] and putative K2PP effectors from plant pathogens [7].

## How can we identify K2PP effectors?

Pro-domains are most often identified due to their absence in the mature, secreted form of the protein, but the ability to identify a pro-domain *in silico* is important in the context of effector biology. Pro-domains are typically located at the N-terminus of proteins and often lack secondary structure features. To search for putative K2PP effectors, we constrained our analysis to the first half of the effector protein sequences and searched for disorder promoting amino acids that proceeded canonical Kex2 cleavage motifs (KR, RR) or the expanded motif (LxxR) (Fig 1B) [7]. Our analysis of 120 secreted effectors from fungal plant pathogens demonstrated that approximately 30% contain a putative Kex2-cleaved pro-domain and included many effectors not previously suspected to be processed [3,14–16]. The candidate list includes effector proteins from necrotrophic, hemibiotrophic, and biotrophic fungi. While experimental validation of many of these remains to be determined, we suggest that K2PP effectors are likely important across a broad range of plant pathogens with diverse lifestyles. The use of more advanced K2PP effector identification methods, combined with experimental validation, will improve our understanding of the importance of K2PP effectors for different fungi.

## What is the role of the pro-domain in K2PP effectors?

Pro-domains are found in a wide range of proteins with diverse biological functions but are most commonly associated with proteases and hormones. There are several canonical functions of pro-domains reported, including localisation, facilitating protein storage, and regulating the activity of the mature protein. The pro-domain itself can also act as an independent biologically active molecule, and they have also been implicated in stabilisation and correct folding of proteins, acting in these cases as an intramolecular chaperone [27,28]. These pro-domains are presumed to work as scaffolds guiding the intermediate, lower energy states, to their native conformation [29].

At present, the role of the pro-domains in the fungal K2PP effectors remains unknown, and it is plausible that multiple, diverse roles exist. In our *in vitro* studies, we found that the pro-domain was required to produce numerous K2PP effectors in their correctly folded form when expressed within a heterologous system [7]. This has also been reported in protein refolding studies of ToxA [30]. While further studies are required, we predict that some K2PP pro-domains possess intramolecular chaperone functions.

## What are the implications and opportunities presented by K2PP effectors?

Identification of a K2PP effector has important implications for investigating their functions. The use of solubility or localisation (florescence) tags and choice of tag location become important considerations for researchers. In addition, many effector studies involve transient or stable expression within the plant hosts. While Kex2-like proteases are conserved across fungi, and homologues identified in other eukaryotes, Kex2-homologues have not yet been identified in plants. This may mean that effectors are not processed in the same way in the plant as they would be in the fungus, which could impact on the outcomes gleaned from these studies. Furthermore, in the age of improved *ab initio* protein structure prediction, ensuring that the mature form of the effector is utilised during prediction could impact the accuracy of the structural model.

## Conclusions

Fungal plant pathogens secrete effector proteins that act to modulate host physiology and promote infection. To date, the grouping of fungal effectors based on sequence features and conserved motifs has been difficult. The observation of the K2PP class of fungal effectors highlights a feature of effector maturation that appears conserved across a wide range of fungal pathogens. K2PP effector identification will have implications for how these effectors are studied in the future. In addition, identification of this conserved K2PP class may provide opportunities to develop novel control strategies to combat fungal pathogens, such as the development of compounds that target and inactivate Kex2.
